# Preparation of MoS_2_ Nanospheres using a Hydrothermal Method and Their Application as Ammonia Gas Sensors Based on Delay Line Surface Acoustic Wave Devices

**DOI:** 10.3390/ma16134703

**Published:** 2023-06-29

**Authors:** Chan-Yu Chung, Ying-Chung Chen, Feng-Renn Juang, Kuo-Sheng Kao, En-I Lee

**Affiliations:** 1 Department of Electrical Engineering, National Sun Yat-Sen University, Kaohsiung 80424, Taiwan; d063010001@student.nsysu.edu.tw (C.-Y.C.); ycc@mail.ee.nsysu.edu.tw (Y.-C.C.); frjuang@mail.ee.nsysu.edu.tw (F.-R.J.); m103010093@student.nsysu.edu.tw (E.-I.L.); 2 College of Semiconductor and Advanced Technology Research, National Sun Yat-Sen University, Kaohsiung 80424, Taiwan; 3 Department of Computer and Communication, SHU-TE University, Kaohsiung 82445, Taiwan

**Keywords:** SAW, MoS_2_, NH_3_, gas sensor, nanosphere

## Abstract

An ammonia sensor based on a delay-line surface acoustic wave (SAW) device is developed in this study by coating the delay line area of the device with a nano-structured molybdenum disulfide (MoS_2_) sensitive material. A SAW device of 122 MHz was designed and fabricated with a pair of interdigital transducers (IDTs) defined on a 128° y-cut LiNbO_3_ substrate using photolithography technologies, and the aluminum IDT electrodes were deposited by a DC magnetron sputtering system. By adjusting the pH values of precursor solutions, molybdenum disulfide (MoS_2_) nanospheres were prepared with various structures using a hydrothermal method. Finally, an NH_3_ gas sensor with high sensitivity of 4878 Hz/ppm, operating at room temperature, was successfully obtained. The excellent sensitivity performance may be due to the efficient adsorption of NH_3_ gas molecules on the surfaces of the nanoflower-like MoS_2_, which has a larger specific surface area and provides more active sites, and results in a larger change in the resonant frequency of the device due to the mass loading effect.

## 1. Introduction

The development and application of industrial technology have improved the convenience of human life, but they have also brought many new challenges, such as negative impacts on air quality and the ecological environment. There are many kinds of pollutants in the air, among which the dust-like particles floating in the air are called particulate matter (PM). When the suspended particulate matter is PM_2.5_ (particle size less than or equal to 2.5 microns), it can penetrate the bubbles in the lungs and cause serious harm to human health as the blood circulates in the body, which cannot be ignored. It has been clarified that the main source precursors of PM_2.5_ are sulfur dioxide, nitrogen oxides, ammonia, etc. [1,2]. The US Occupational Safety and Health Administration (OSHA) has declared that the acceptable average ammonia exposures time is 8 h at 25 ppm and 15 min at 35 ppm [3,4]. If the human body strongly inhales high concentrations of NH_3_, various symptoms such as tearing, coughing, and dyspnea will appear, and death can be caused under extremely high concentrations of ammonia [5,6]. 

Ammonia gas (NH_3_) is commonly used in industrial manufacturing processes. However, when the concentration of NH_3_ in the air reaches a certain range, of about 15% to 28%, it is explosive. Many kinds of NH_3_ sensors using various sensitive materials have been developed. For example, metal-oxide sensors, which are current research hotspots, have the advantages of simple operation, low cost, and fast response, but complex synthesis processes and higher operating temperatures are required for better performance, and because of high power needed, their application in the Internet of Things is bound to be limited [7,8,9,10,11]. Therefore, there is an urgent need to study high-performance NH_3_ sensors operating at room temperature for immediate preventive measures under adverse conditions.

In this study, a SAW device was employed to perform as an ammonia sensor using novel nanostructured molybdenum disulfide (MoS_2_) as the sensitive layer. In the application of gas sensors, surface acoustic wave devices have the advantages of high sensitivity, low detection limit and operation at room temperature, which can be used to measure the trace concentrations [12,13,14]. In addition, the frequency responses of SAW sensors can support wireless sensing, and also have outstanding performance at room temperature [15]. 

The resonant frequency f of a surface acoustic wave device is related to the spacing of the interdigital transducer (IDT) electrodes and the wave velocity, with the relationship of f = υ/λ where υ is the wave velocity, λ is the wavelength, and f is the resonant frequency. The 4 times the line width of the electrode (d) can excite the acoustic wave, with a wavelength of λ = 4d. In the design of a SAW gas sensor, the delay line area of the SAW device is coated with a sensitive thin film, and the gas molecules are adsorbed through the sensitive thin film to produce physical changes, which will change the resonant frequency of the SAW device [16,17]. 

Molybdenum disulfide (MoS_2_) has a large band gap, a layered structure, semiconductor characteristics, etc. It is an excellent gas-sensing material, with high sensitivity and selectivity when applied as a sensor [18,19]. In addition, studies have shown that the shape, size, and specific surface area of gas-sensing materials also have a great influence on the gas-sensing performance [20]. Therefore, it is of great significance to study the gas-sensing properties of MoS_2_ with different morphologies for the design of sensor structures. In this study, three kinds of ammonia sensors with different MoS_2_ morphologies were constructed based on the hydrothermal method, and the gas-sensing properties of MoS_2_ with three different morphologies for ammonia gas were studied.

## 2. Materials and Methods

### 2.1. Preparation of Molybdenum Disulfide Nanospheres

**Figure 1:**
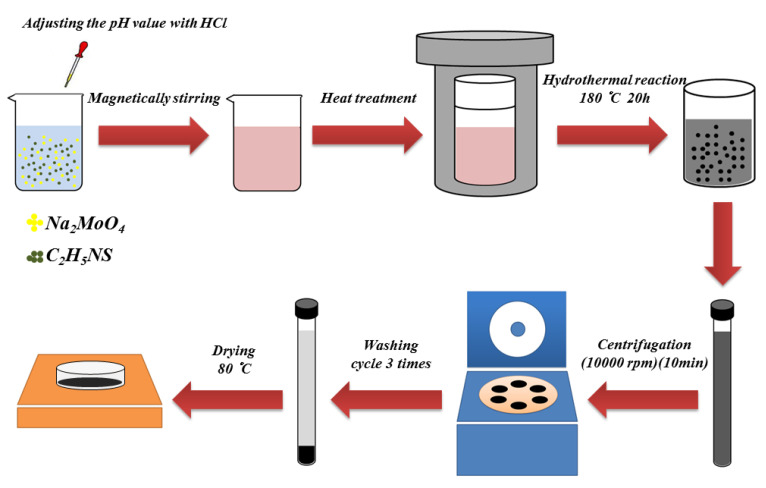
Process flow of the hydrothermal method to prepare MoS_2_ nanospheres.

In this study, MoS_2_ nanospheres, prepared with a simple hydrothermal method, were used as the sensitive materials of the surface acoustic wave gas sensors. Briefly, Na_2_MoO_4_ (0.05 M) and C_2_H_5_NS (0.15 M) were added to 40 mL of deionized (DI) water and stirred for 30 min to fully dissolve. In order to obtain different MoS_2_ morphologies, various amounts of HCl were added to adjust the pH values of the solutions, with pH1, pH3, and pH5, respectively, and were then stirred for 10 min to form uniform precursor solutions. Subsequently, the precursor solution was poured into 100 mL of Teflon liner, then loaded into an autoclave reactor and heated to 180 °C in a high-temperature furnace for 20 h. Finally, the obtained powders were washed several times by centrifuge with ethanol and deionized water. After drying at 80 °C for 6 h on a heater platform, various morphologies of MoS_2_ nanospheres were obtained. The process flow of the hydrothermal method is shown in Figure 1. 

### 2.2. Fabrication of Surface Acoustic Wave Devices

**Figure 2:**
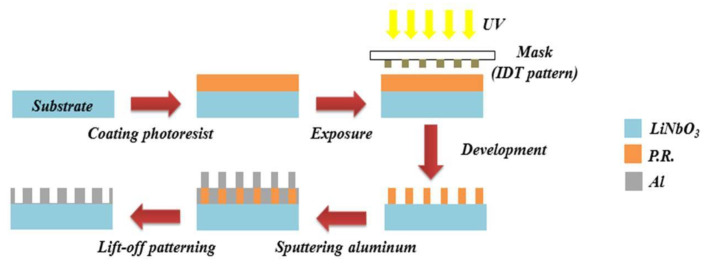
Fabrication processes of a surface acoustic wave device [21].

LiNbO_3_ is a crystal that integrates multiple effects such as piezoelectricity, electro-optic, and nonlinear optics, and has the advantages of high temperature resistance and corrosion resistance. In this study, 128° y-cut LiNbO_3_ was adopted as the piezoelectric substrate with a wave speed of ≈3900 m/s. The line width of the interdigital transducer (IDT) electrodes of 8 µm was designed to excite the surface acoustic wave with a wavelength of 32 µm, which resulted in a resonant frequency of 122 MHz. Figure 2 shows the fabrication processes of a SAW device. Firstly, the 128° y-cut LiNbO_3_ substrate was cleaned according to the RCA cleaning processes, and then the photoresist (AZ1500) (1.2 µm) was coated on the substrate using a spinning coater, and heated on a heater platform for soft baking, and then exposed and developed through the usual photolithography processes. In this study, a dual-target DC sputtering system was used to deposit Ti (20 nm) and Al (100 nm) as IDT electrodes, in which the titanium metal film was used as an adhesive layer. The advantage of the double-target sputtering system is that it can continuously deposit multi-layer films without restarting the vacuum chamber, avoiding pollution or oxidation on the films. Therefore, a high-quality SAW device can be obtained through controlling the film quality. Finally, the lift-off process was carried out by immersing the substrate into the acetone solution and shaking using an ultrasonic washing machine to remove the photoresist and unnecessary films to complete the SAW device.

### 2.3. Sensor Fabrication

**Figure 3:**
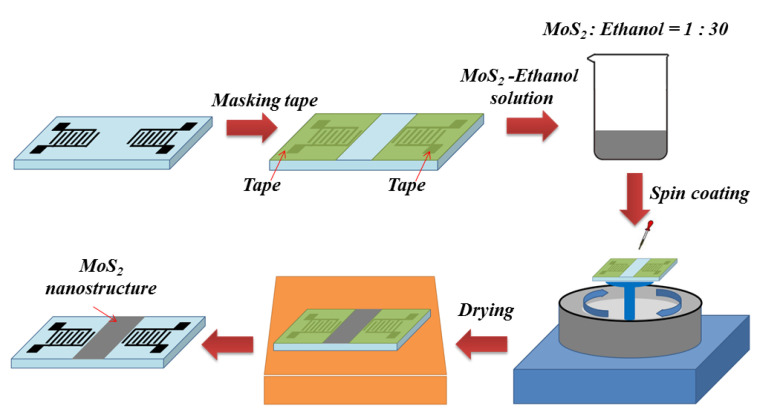
Fabrication flow chart of a SAW gas sensor based on MoS_2_ nanospheres.

**Figure 4:**
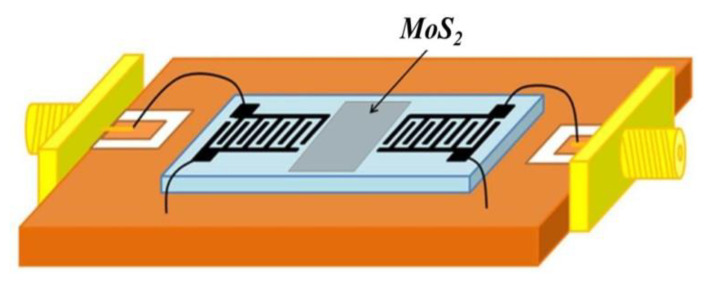
Schematic of the complete ammonia gas sensor.

In this study, a SAW delay line structured device with a resonant frequency of 122 MHz was successfully fabricated on the 128° y-cut LiNbO_3_ substrate. Then, the MoS_2_ nanospheres, as the sensitive materials, were spin-coated on the delay line area of the SAW devices. Figure 3 shows the preparation processes of the sensitive layer of the sensor. First, the IDT electrodes of the SAW device were covered with tape. Then, the MoS_2_ powders were mixed with absolute ethanol at a weight ratio of 1:30, and stirred for 20 min. Then, the MoS_2_ solution of 1 mL was dropped on the delay line area using a precision dropper, and spun at 1200 rpm for 30 s. Finally, the substrate and the sensitive materials were dried at 90 °C for 6 min. After removing the tape, the sensor device was fabricated. Figure 4 is a schematic diagram of the complete ammonia gas sensor. The SPB-U668 aluminum wire bonder was adopted to connect the SAW sensor and the microstrip line, according to the design rules of the coplanar waveguide (CPW) microstrip line and the impedance calculation of the 50 ohm (PCB) board.

### 2.4. Material Analysis and Sensor Measurement

The nanostructures of MoS_2_ will affect the properties of the sensor devices. Therefore, scanning electron microscopy (SEM, JEOL-6700 field emission SEI/BEI type, JEOL, Ltd., Tokyo, Japan), energy-dispersive X-ray spectroscopy (EDX, JEOL, Ltd., Tokyo, Japan), and X-ray diffraction (XRD, Bruker, Billerica, MA, USA) were used to analyze the nanostructures and atomic ratio of MoS_2_ under different hydrothermal conditions. The frequency response of the SAW sensor was measured using the P9372A Keysight Streamline USB Vector Network Analyzer (Keysight, Santa Rosa, CA, USA). 

Gas sensing measurements were performed on a static gas sensing measurement system at room temperature. During the test, a 25% ammonia solution was carried by N_2_ gas and injected into the vaporizer through a micro-syringe, then mixed with air in a 3 L test chamber to prepare NH_3_ gas with various concentrations of 5–50 ppm. Finally, the desired concentration of NH_3_ gas was introduced into the measurement chamber, and the frequency response of the sensor exposed to different concentrations of NH_3_ gas was recorded by the network analyzer.

## 3. Results and Discussion

### 3.1. Performance of the Designed Surface Acoustic Wave Device

The performance of a SAW device can be defined by its figure of merit (*FoM*), as in Equation (1).

(1) $$FoM=Q\times K_{}^{2}$$

where *Q* is the quality factor, and *K*^2^ is the electromechanical coupling factor of the SAW device. Besides, *Q* and *K*^2^ can be derived from the following Equations (2) and (3).

(2) $$Q=\frac{f_{0}}{f_{H}-f_{L}}$$

(3) $$K_{}^{2}=\frac{\pi }{4N}\times \frac{Ga}{B}$$

where *f*_0_ is the center frequency of the SAW filter, *f_H_* and *f_L_* are the upper and low 3-dB frequencies of the SAW filter, respectively. *B* is the radiated susceptance; *Ga* is the radiated conductance; and *N* is the IDT pair number.

In practical applications, it is necessary to pursue SAW devices with high electromechanical coupling factors (*K*^2^) and quality factors (*Q*). However, there are some trade-offs between these two parameters. Therefore, *FoM* can better demonstrate the advantages and disadvantages of the device during the design process of SAW. In this study, the fabricated SAW device exhibited a *Q* factor of 180, *K*^2^ of 3.29 % and *FoM* of 5.922.

### 3.2. Characteristic Analysis of Molybdenum Disulfide 

**Figure 5:**
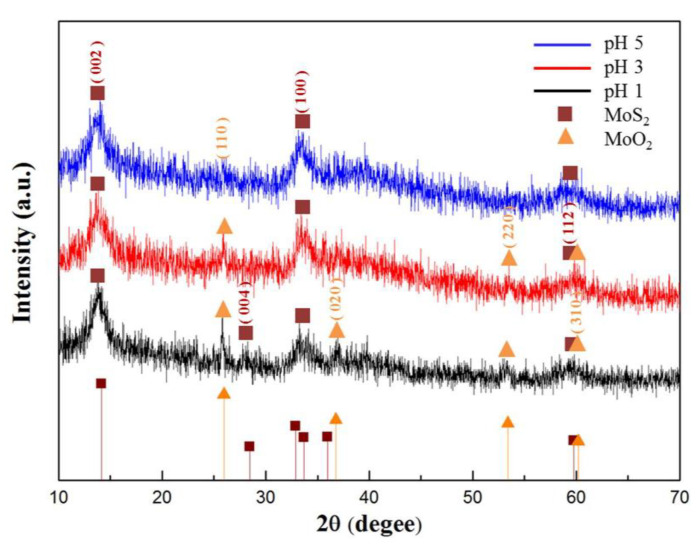
X-ray diffractions of MoS_2_ samples obtained from precursor solutions with different pH values.

In this experiment, by adjusting the pH values of the precursor solutions, various MoS_2_ nanospheres were successfully prepared using the hydrothermal method. It is demonstrated that HCl will play an important role in the synthesis of MoS_2_ [22]. Without HCl, the pH value of the precursor solution was about 6, and no MoS_2_ powder was produced at this situation. As HCl was added into the precursor solution, MoS_2_ appeared without any byproduct at pH value of 5. However, as the concentration of HCl increased further, the byproduct MoO_2_ appeared at pH values of 3 and 1. The X-ray diffractions of obtained MoS_2_ samples with different pH values were shown in Figure 5. It can be inferred that HCl may enhance the formation of MoS_2_ and MoO_2_.

**Figure 6:**
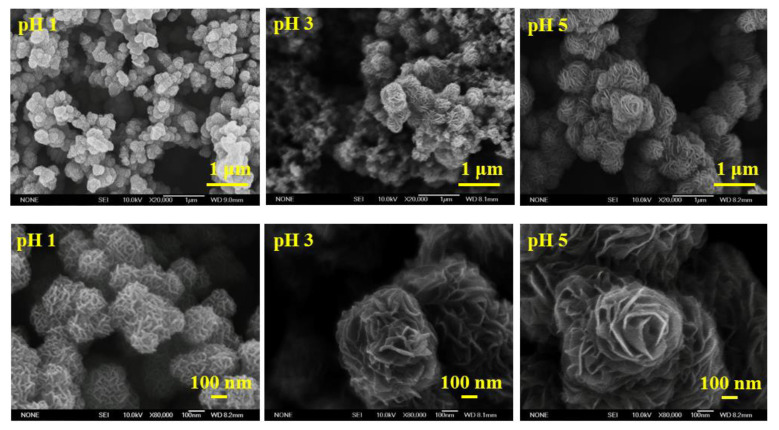
SEM images of MoS_2_ microstructures obtained from precursor solutions with different pH values.

The concentration of HCl in the reaction solution not only affects the formation of MoS_2_, but also has a great influence on the microscopic morphology. From the SEM images in Figure 6, it can be found that the nanospheres are formed by flake agglomeration. As the pH value increases, the flake structure becomes more obvious. When the pH value was 5, the structure resembled a nanosphere of a rose. The experimental results show that the morphological control of the product could be achieved by adjusting the concentration of HCl in the reaction solution. 

From the EDX analysis, it can be clearly observed that there were only Mo and S elements in the MoS_2_ samples without impurities. Moreover, as the pH value approached neutral, the atomic ratio of Mo and S approached 1:2, as shown in Table 1.

### 3.3. Analysis of Ammonia Gas Sensor 

**Figure 7:**
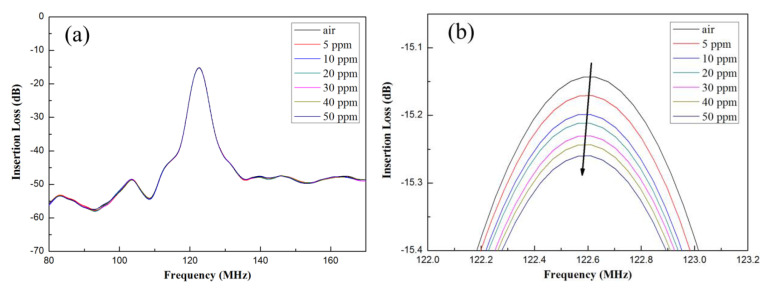
(**a**) Frequency variations at different ammonia concentrations for sensor−1. (**b**) Zoomed in near the region of 122 MHz resonant frequency.

**Figure 8:**
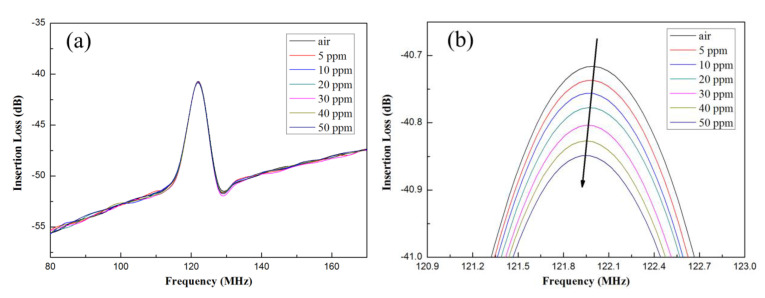
(**a**) Frequency variations at different ammonia concentrations for sensor−2. (**b**) Zoomed in near the region of 122 MHz resonant frequency.

**Figure 9:**
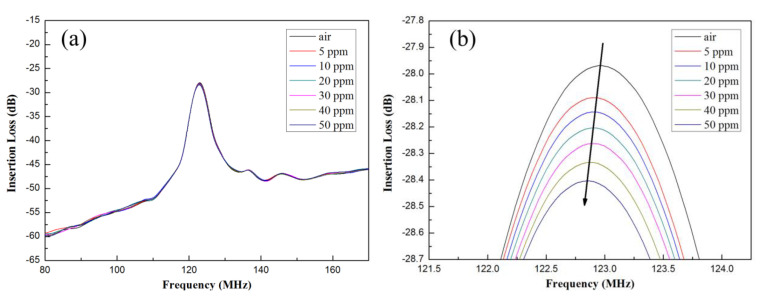
(**a**) Frequency variations at different ammonia concentrations for sensor−3. (**b**) Zoomed in near the region of 122 MHz resonant frequency.

The MoS_2_ nanospheres obtained from various precursor solutions with pH values of pH1, pH3, and pH5 were coated on the delay line area as the sensitive layers of the SAW gas sensors, and those were defined as sensor−1, sensor−2, and sensor−3. As the gas sensors were exposed to different concentrations of NH_3_, variations in the frequency response could be observed and shown in Figure 7a for sensor−1, 8a for sensor−2, and 9a for sensor−3. For clarity, the sensor response near the resonant frequency of 122 MHz was zoomed in further, as shown in Figure 7b, Figure 8b and Figure 9b.

**Figure 10:**
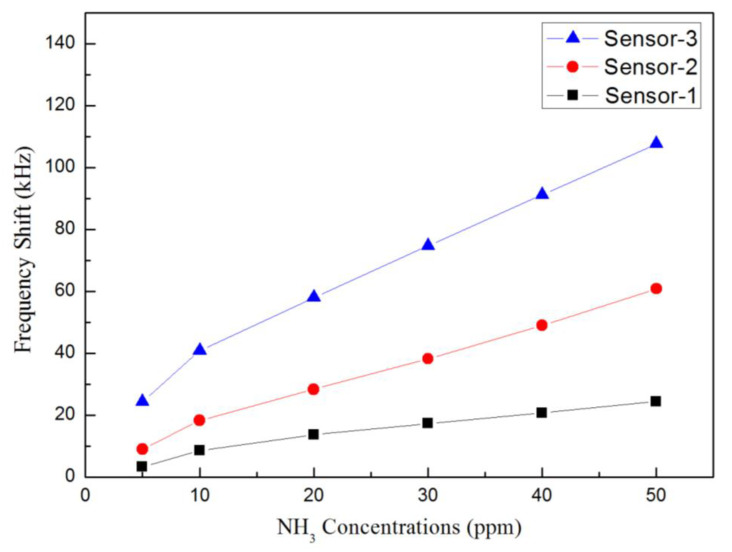
The frequency shifts of the fabricated sensors towards NH_3_ gas concentrations.

The original resonant frequency of all sensors without NH_3_ gas was around 122 MHz, whereas it shifted toward lower frequencies as the concentration of NH_3_ gas increased. The frequency shifts for the three sensors are shown in Figure 10. Sensitivity is an important indicator of sensor performance, which refers to the ratio of sensor output variation to measured input variation; its calculation formula is as follows:

(4) $$S=\frac{\left|f_{0}-f_{gas}\right|}{∆c}$$

where *S* is the sensitivity, ∆c is the change of gas concentration, $f_{0}$ is the frequency of the gas sensor in air, and $f_{gas}$ is the frequency at which the gas sensor detects the measured gas. From Equation (4), the calculated sensitivities were 1402 Hz/ppm for sensor−1, 2816 Hz/ppm for sensor−2 and 4878 Hz/ppm for sensor−3, respectively. The detection limit could be as low as <1ppm.

In the SAW sensor, the resonant frequency change (Δ*f*) may be related to mass loading or acoustoelectric interactions, or elastic changes when interacting with target gas molecules [23]. However, the lower value of the electromechanical coupling factor for 128° y-LiNbO_3_-based SAW sensor is expected to result in a negligible contribution due to the acoustoelectric effect [24]. Therefore, the existence of acoustoelectric interaction can be ruled out in this case. Furthermore, it is essential to point out that mass loading causes the resonant frequency to move towards the lower end (i.e., decreasing the resonant frequency), while the elastic changes cause the resonant frequency to move towards the higher end (i.e., increasing the resonant frequency); that is, the material elasticity can also be ruled out and the change was negligible [24]. From the results obtained, it can be concluded that the frequency shift is owing to the mass loading effect.

The mass loading effect of a sensor can be expressed by the following formula [23,24,25,26]:

(5) $$∆f=(k_{1}+k_{2})\times f_{0}^{2}\times ∆\rho s$$

where *k*_1_ (−3.775 × 10^−8^ m^2^ s kg^−1^) and *k*_2_ (−1.73 × 10^−8^ m^2^ s kg^−1^) are the material constants of 128° y−cut LiNbO_3_ substrate. Δ*ρs* is the density change of the sensing layer of the SAW sensor after being exposed to ammonia. Note that both *k*_1_ and *k*_2_ are negative, so a positive change in Δ*ρs* results in a negative value in Δ*f* [27,28,29].

In this study, sensor−3, prepared with MoS_2_−pH5 as sensitive material, exhibited the best performance as an ammonia sensor. It is known from the literature that nanoflower−like MoS_2_ has the highest specific surface area, and it is speculated that a larger specific surface area can provide more active sites, thereby obtaining higher ammonia gas−sensing response performances [30,31,32,33]. The resonant frequency of the SAW sensor shows a downward shift as the concentration of NH_3_ increases. The frequency variation for 5–50 ppm NH_3_ gas is shown in Table 2 for sensor−3, from which the sensitivity was calculated to be 4878 Hz/ppm. Compared with other works in the literature, this study showed an excellent sensitivity performance [34,35,36,37,38], as shown in Table 3.

## 4. Conclusions

In this study, SAW devices of 122 MHz were fabricated using the piezoelectric 128° y−cut LiNbO_3_ as the substrate. MoS_2_ nanospheres were prepared using the hydrothermal method and coated on the delay line area of SAWs. Finally, an NH_3_ gas sensor with a high sensitivity of 4878 Hz/ppm was successfully obtained. The reason for the improved sensitivity was due to the efficient adsorption of the target NH_3_ gas molecules on the surfaces of the nanoflower−like MoS_2_ sensitive layer, which had a larger specific surface area and provided more active sites, resulting in a more significant change in the resonant frequency of the device due to the mass loading effect.

## Figures and Tables

**Table 1:** EDX analysis of MoS_2_ nanospheres obtained from various precursor solutions.

*Precursors*	*pH1*	*pH3*	*pH5*
***Element***	*Atomic %*	*Atomic %*	*Atomic %*
***S***	61.32	65.24	67.57
***Mo***	38.68	34.76	32.43
***Atomic ratio (S/Mo)***	1.59	1.88	2.08

**Table 2:** Resonant frequency and frequency shifts with different concentrations of NH_3_ for fabricated MoS_2_/SAW sensor−3.

*Concentration of NH_3_* *(in ppm)*	*Resonant Frequency* *(in MHz)*	*Shift in Frequency* *(in kHz)*
0	122.956197	Reference
5	122.931808	24.389
10	122.915316	40.881
20	122.898117	58.08
30	122.881384	74.813
40	122.864918	91.279
50	122.848529	107.668

**Table 3:** Comparisons of NH_3_ sensing performance of SAW−based sensors with various sensing materials.

*Working Frequency (MHz)*	*Sensing Material*	*Sensitivity (Hz/ppm)*	*Ref.*
100	SnO_2_/Co_3_O_4_	3.33	[34]
200	SiO_2_−SnO_2_	210	[35]
200	SiO_2_−TiO_2_	2000	[36]
200	AlO(OH)	154	[37]
200	TiO_2_	500	[38]
122	MoS_2_	4878	Present work

## Data Availability

Not applicable.
